# Pharmacological factors across cultures

**DOI:** 10.1192/j.eurpsy.2026.10399

**Published:** 2026-07-31

**Authors:** D. Bhugra

**Affiliations:** Kings College London, London, United Kingdom

## Abstract

**Abstract:**

Pharmacological factors and challenges across Cultures

Cultures are important factors in defining what is normal and what is abnormal. These abnormal behaviours determine where help is sought from. Cultures influence explanatory models and also how individuals express and deal with distress – whether it is physical or emotional. Culturally influenced are accepted by healthcare workers if they are from same culture. Cultures and society dictate health resources as well as encourage individuals to follow certain pathways into care and how they accept or reject health care. Cultural variations in presentation of various psychiatric disorders have been studied, but not treatment responses as widely.

In addition to pharmacokinetics and pharmacogenetics, otherfactors such as body weight, dietary habits and simultaneous use of alternative and complementary medicines will affect how the role of medication is perceived and accepted which then affect and pharmacodynamics. All these factors play a major role in acceptance or rejection of pharmaceutical agents due to potential side effects and resulting treatment outcomes across cultures.

In various cultural settings, patients and their carers have varying expectations and responses to medication. This talk will explore various factors which affect acceptance of psychiatric medications across different cultural groups.

**Disclosure of Interest:**

None Declared

